# Provincial Medical and Surgical Association

**Published:** 1838-10

**Authors:** 


### PART FOURTH.

### Medical Intelligence.

### PROVINCIAL MEDICAL AND SURGICAL ASSOCIATION.

The Sixth Anniversary Meeting of this Society was held on Wednesday and Thursday, the 18th and 19th of July, at Bath. On Wednesday morning, at twelve o'clock, a meeting of the council took place, at the rooms of the Bath Royal Literary and Scientific Institution, when the financial and other routine business of the Society was transacted, and preparations made for the general meeting. This took place at seven o'clock in the evening, and was very numerously attended. Dr. Boisragon, the late President, having resigned the chair, it was taken by Dr. Barlow; both gentlemen addressing the meeting in elegant and appropriate speeches. Dr. Hastings, the Secretary, read the Annual Report of the Council, which represented the Society to be in a very flourishing condition, both the funds and the number of members being annually increasing. The actual number of members enrolled previously to the meeting was 1080; and no doubt the number was considerably increased before the close of the reunion at Bath. Besides the ordinary routine business of voting resolutions of thanks, &c., much interesting discussion on various important subjects took place, and committees were appointed to carry out the views of the Association. The two principal of these were, one on the subject of Quackery, and one on Small-pox and Vaccination; both subjects of much interest, and the latter of surpassing importance. At this sitting, also, the following gentlemen were unanimously elected HONORARY CORRESPONDING MEMBERS, on the proposition of Dr. Forbes:

*For Russia:* F. C. Markus, M.D., chief physician to the Galitzin Hospital, Moscow; George Lefevre, M.D., physician to the British embassy, St. Petersburgh.

*For Sweden and Norway:* D. Holst, M.D., professor of Medicine in the Royal Frederick's University, Christiania.

*For Denmark:* C. Otto, M.D., professor of Pharmacology and Forensic Medicine in the University of Copenhagen.

*For Austria:* Burkard Eble, M.D., librarian of the Josephine Academy, Vienna.

*For Holland:* J. L. Schroeder van der Kolk, M.D., professor of Anatomy and Physiology in the University of Utrecht.

*For France:* E. C. A. Louis, M.D., physician to La Pitié, &c., Paris; G. Andral, M.D., professor of Pathology in the Faculty of Medicine, Paris.

*For Italy:* Carlo Francisco Bellingeri, M.D., president of the Medical Faculty in the University of Turin, &c.; Maurizio Bufalini, M.D., professor of Clinical Medicine at the Hospital Santa Maria, Florence.

*For Portugal:* Antonia Jose de Lima Leitaõ, M.D., physician to the Hospital San Lazaro, at Lisbon.

*For the United States of America:* John C. Warren, M.D., professor of Anatomy and Surgery, Harward University, Boston; Robley Dunglison, M.D., professor of Materia Medicine, &c., Jefferson College, Philadelphia.

*For the East Indies:* W. B. O. Shaughnessy, M.D., professor of the Institutes of Medicine in the Medical College, Calcutta.

*For the Brazils:* Luis Vicente De Simoni, M.D., secretary of the Imperial Academy of Medicine of Rio Janeiro.

*For Mexico:* Guillermo Schiede, M.D., member of the Academy of Medicine, Mexico.

*For Australia:* E. C. Hobson, M.D., naturalist to the colony of Van Diemen's Land, Hobart Town.

After the transaction of other necessary business, the meeting adjourned till the following morning; when, shortly after nine o'clock, the members of the Association breakfasted together at the Pulteney Hotel, Sydney Gardens. Arrangements were entered into at the breakfast for the immediate execution, and presentation to Mrs. Hastings, of the portrait of Dr. Hastings; two hundred pounds being already subscribed for that purpose. The artist is not yet decided on.

At one o'clock the members again met at the lecture-room of the Institution, when various interesting subjects were discussed, and a good deal of business done.—Dr. Gregory, of London, made some observations on the varieties and best mode of preserving vaccine lymph; and presented some specimens to the meeting.

Mr. Thompson read a paper on "a peculiar Affection of the Uvula," which will be inserted in the next volume of the Transactions. Dr. Johnstone Aitkin, of Poole, read a communication respecting the use of the sea-plant called *Fucus esculentus*, or "tangle," in the room of the more expensive and less suitable gum bougies, in cases of stricture of the rectum and urethra.

Dr. Theodore Boisragon produced some drawings, executed by himself, of various parts of the human skeleton, and announced his determination to publish them at a very reduced price. We have no doubt of their usefulness to practitioners.

Dr. Conolly, of Cheltenham, then read the Report of the Benevolent Fund Committee, which showed the increasing utility of its operations. The receipts for the past year were—balance, 34*l.* 4*s.*; donations, 84*l.* 6*s.* 6*d.*; subscriptions, 115*l.* 3*s.* total, 233*l.* 13*s.* 6*d.* Expenditure, 89*l.* 5*s.* Total balance, 144*l.* 8*s.* 6*d.* This balance belongs to what is called the "permanent fund," and is not available to the general purposes of the Society, but is applied, in the shape of annuities, to widows and orphans. Much interest was excited by the report and consequent discussions; which, we doubt not, the fund will feel the benefit of before the next meeting of the Association.

Much other business was transacted, but not of a kind to interest the general reader, and the proceedings of the morning were concluded by the delivery of the annual Retrospective Address, by Dr. Malden, of Worcester. This was a very learned and eloquent production, as might be expected from the well-known talents of the author; but, as we shall have an opportunity of noticing it when it is published in the Transactions, we shall give no further account of it at present.

Between six and seven o'clock, the members and their friends dined together at the Town-hall; Dr. Barlow in the chair, supported on the right by the mayor of Bath, and on the left by the Rev. William Marshall, his chaplain: vice-presidents, William Tudor and George Norman, Esqs. The dinner, dessert, and wines, were of the first quality. An excellent band was in attendance; and we can truly state, in newspaper phrase, that the evening was spent in the utmost harmony and enjoyment.

The next anniversary meeting of the Association is to be held at Liverpool, under the presidency of Dr. Jeffreys; and Dr. Symonds, of Bristol, is appointed to prepare the Retrospective Address.

We regret that our limits prevent us from giving, in greater detail, an account of this interesting meeting; but we must find room for the short but admirable speech of Dr. Barlow, on assuming the chair. This we copy from the columns of a newspaper, (the Worcester Journal,) which gave, at the time, an excellent and accurate report of the proceedings. The flattering manner in which Dr. Barlow was received by the Association was in accordance with the high character which he holds in the profession; distinguished, as he is, for his literary and scientific acquirements, his great practical skill, his honorable bearing towards all his brethren, and his active and unbounded benevolence to the sick poor.

### *Dr. Barlow's Address.*

"Gentlemen: In entering on the office which your kindness has assigned to me, my first agreeable duty is to bid you all heartily welcome to this our ancient city, which was never more signally honoured than it is on the present occasion. Cultivated talent and moral worth, especially when combined, must ever receive the respect and regard of all who are capable of appreciating them. For both, our revered profession has ever been eminent; and when they who, even amongst its members, distinguish themselves by pressing forward in the career of humane and enlightened endeavour, assemble in such numbers as I rejoice to see now around me, for the purpose of cultivating still further their divine art, and promoting the best interests of humanity,—their presence must confer honour on any place which is graced by such an assemblage. It is not my design, gentlemen, to trespass long on your time or attention in the address from the chair, with which the customs of our Association require me to open the present session. To do so would be an abuse of the privilege which my present situation confers, and prove only an irksome delay of the far more interesting matter which will be speedily submitted to your consideration. In each successive year some change takes place in the circumstances under which your president addresses you. Heretofore, and until the designs for which the Association was instituted had become generally understood, it was the duty of your presidents, in their respective discourses, to dwell on those designs, and the evidences of their fulfilment, so as to make the nature, scope, tendency, and progressive realization familiar to all concerned. Happily this is no longer needed; for the years that have elapsed since we first assembled to found this Association, and the wide diffusion of our Reports and Transactions, have made these designs fully known; while extension of the Association, which, in respect both of numbers and space, has advanced with a rapidity which I may say is unexampled, furnishes assurance the most unequivocal of their being justly appreciated. Were further proof of this needed, the assemblage which I now see before me, congregated from almost every part of the kingdom, must suffice to carry conviction to the most sceptical. And here, gentlemen, I will remark that, so long as we display such evidence of zealous and harmonious cooperation, we may be content to pursue the direct and even tenour of our way, whatever the opposition we may chance to encounter; and, cheered by the consciousness that, so far as our abilities extend, we are pursuing laudable objects from pure motives, may safely disregard objections, such as only ignorance or misconceptions of our designs could urge against us. Practical details and statistical elucidations you will have abundantly in the ulterior proceedings of the present meeting: on all such it would be vain and idle for me to dwell. I prefer, therefore, during the few moments to which my present trespass shall be limited, to direct your attention to those considerations which admit not of statistical exposition, yet which are not the less valuable from requiring to be addressed rather to the mind's eye than to our actual perceptions.—The main objects for which we are associated, as stated in our fundamental constitution, are the advancement of medical science, and the maintenance of the honour and respectability of the profession. These objects are intimately connected; for, unless science be diligently and effectively cultivated, the honour and respectability of the profession would rest on a very slight foundation; and, unless the honour and respectability were otherwise maintained, on the high ground of moral integrity and liberal sentiment, no advance in science could vindicate its claim to that high estimation in which it has through ages been held, and which, I trust, it will ever, even with sensitive jealousy, preserve. The feelings of the sensitive Roman, who would not that his wife should be even suspected of error, are to be commended; and, with similar feelings, it should be our care so to conduct the proceedings of our Association, that not even the suspicion of selfish or sinister designs should attach to us. To the cultivation of medical science our endeavours have been hitherto directed, with an earnestness and steadiness of which it becomes not me here to speak. However little these endeavours may have hitherto produced, they have at least been exerted with a zeal worthy of the cause which called them forth. My present purpose, however, is not to dilate on these efforts or their fruits, but to impress on you all that they who would judge of the value of our Association, even by the efforts already made, or the products which have resulted from them, would form but a very imperfect estimate of the benefits which our Association is conferring, and which it cannot fail eventually to realize. It has been asked, and in a depreciating tone and unfriendly spirit, what have we done? The very question conveys to me the conviction that the party proposing it has no adequate conception of the subject on which he affects to seek information. No one, really imbued with the love of science or the spirit of truth, would even form the conception of judging us by so crude and inadequate a test. It is, no doubt, true that fruits should be the proof by which modes of cultivation should be judged; but, surely, not till time be given for seeds to germinate and plants to fructify. In our cultivation of medical science, it surely cannot be barren of fruits when upwards of one thousand energetic members of a liberal and enlightened profession are incited by the inspiriting stimulus which this Association supplies, to exert their best faculties and most earnest efforts for investigating those truths of nature which it has ever been the object and aim of our profession to explore. In the activity thus aroused, there is an ample assurance that the energies so called forth will not be unprofitable; that to the seed thus sown may we look with full confidence for a rich and abundant harvest. I care not, gentlemen, how slowly this harvest advances; it being enough to satisfy me that it is advancing. I am not impatient for brilliant discoveries, such as the history of science has shown to occur only at intervals few and far between. Science is ever of slow advance; if this is to be judged by the sudden bounds by which consummate genius starts ahead of contemporary talent marking epochs in the history of the science. But it is ever steadily progressive, if we note the slow but sure, the humble, unpretending, but diligent and unwearied labour with which its ordinary votaries endeavour to extend it. Among these humble labourers do we class ourselves; with the merit attaching to such labour we will be content, and on the result of such labour we are satisfied to rely. Should it fall within the inscrutable designs of Providence that some master-mind should spring up amongst us,—some heaven-born genius, destined to achieve the performances and equal the eminence of a Newton or a Harvey, we shall gratefully hail the distinction, assuming only the humble merit of having used our best endeavours to incite and cherish such transcendant talent. But, gentlemen, in the ordinary pursuit of our objects we look not for such results; and on the diligent exercise of ordinary talents are we content to rest our claims for commendation, encouragement, and support. I am led to submit these views to you, gentlemen, believing them to be those of truth and sober reason; for, while I would deprecate all extravagant anticipations and vain boastings, I conceive it essential to the steady progress of our combined exertions that we neither undervalue what we have done nor form an incorrect estimate of what our conjoined labours are capable of effecting.

"On the second head of my present address,—that, namely, which relates to the maintenance of the honour and respectability of the profession, I shall be very brief; for this honour and respectability must ever flow, not from self-elating pretensions or arrogant claims to consideration, but from the professional skill and moral worth of the individual members. As the aggregate of parts constitutes the whole, so must the maintenance of honour and respectability by each individual member of our Association ensure, beyond the possibility of failure, the continuance of these long-enjoyed attributes to the collective body: and, when I consider the high moral qualities which the members of our body on all occasions display, the talents they evince, and the zeal they manifest,—to all of which even the brief records of our Association already bear ample testimony,—I can entertain no fears of our ever, as a profession, descending from that high moral eminence on which the opinions of the world, and the express declaration of several of the sagest and most acute observers of human nature, have for ages placed us. On the conduct of our individual members I confidently rely for preserving unsullied that reputation which the profession has hitherto maintained. I would here only impress one caution respecting those measures in which we may in our corporate capacity be called on to engage. Corporate proceedings are subject to the same laws, and tried by the same test, as individual conduct. All flow from motives more or less elevated or debased; and, where the higher motives can be brought to bear on any point, those of a lower class should never be suffered to prevail. The human mind is endued with various faculties, all suited to this our transient and probationary state of existence. We have various animal incitements to urge us to whatever is necessary for the continuance and enjoyment of life; but we have also moral faculties to control the lower propensities, and judge those actions to which our animal nature may impel us. It too often happens that the dictates of the animal propensities assume the garb and exercise the authority of the moral sentiments; thus misleading into error many who, under the delusion, are scarcely conscious of doing wrong. But an honest scrutiny of motives, an ingenuous reference of each of these to the moral or animal feeling in which it originates, can never fail to detect the deception, and guide into the right path all who desire to pursue it. These comments would here be impertinent, but that they serve to indicate the criterion by which each corporate act of our Association should invariably be judged. Whenever the act is such as all the higher moral feelings of our nature clearly sanction and approve, let us fearlessly perform it, regardless of all that imperfect conception or timidity may urge against it. On the contrary, whenever an animal or selfish impulse can be traced among the influencing motives, however slight the degree in which this may be intermingled, let us ever pause until we separate the moral motives from the dregs which vitiate them, and restore the higher sentiments to that supremacy with which the Creator has endowed them. I have assimilated corporate acts to individual in the motives which incite them, and the tests by which they are to be tried. But there is one difference which deserves to be noticed as important in our present view of the subject. Corporate acts are less redeemable than individual, and therefore require to be still more carefully guarded from error. Hitherto we have been guarded in our corporate measures; and to ensure a continuance of the same circumspection is the end to which the few remarks which I have now offered are directed. With these I now conclude my present trespass on your time and patience, respectfully yet earnestly exhorting the several members of our excellent Association to apply the energies of their own minds to both the important topics to which I have referred. It will gratify me if what I have ventured thus cursorily to submit to them shall, on deliberate reflection, receive the sanction of their own judgments; and still more if, to the sentiments to which I have given utterance, their own feelings shall be found to respond. So far as my judgment and feelings are capable of guiding me, I would say, in cultivating medical science disdain not, through vain aspirations for profound theories or dazzling generalizations, that patient observation of nature and diligent collection of accurate facts, from which all true theory must be derived, all sound generalization deduced; and, in upholding the honour and respectability of the profession, let the measures we collectively sanction ever bear the impress of that high-toned moral feeling which has so long distinguished our profession, and by which its true interests require us ever to abide."

---

